# Draft Genome Sequences of Two Protease-Producing Strains of *Arsukibacterium*, Isolated from Two Cold and Alkaline Environments

**DOI:** 10.1128/genomeA.00585-15

**Published:** 2015-06-04

**Authors:** Jeanette E. Lylloff, Lea B. S. Hansen, Morten Jepsen, Peter F. Hallin, Søren J. Sørensen, Peter Stougaard, Mikkel A. Glaring

**Affiliations:** a Department of Plant and Environmental Sciences, University of Copenhagen, Frederiksberg, Denmark; b Department of Biology, University of Copenhagen, Copenhagen, Denmark; c Novozymes A/S, Bagsværd, Denmark

## Abstract

*Arsukibacterium ikkense* GCM72^T^ and a close relative, *Arsukibacterium* sp. MJ3, were isolated from two cold and alkaline environments as producers of extracellular proteolytic enzymes active at high pH and low temperature. This report describes the two draft genome sequences, which may serve as sources of future industrial enzymes.

## GENOME ANNOUNCEMENT

Cold and alkaline environments are very rare on Earth, and knowledge regarding the organisms that thrive in such polyextreme environments is limited. The submarine ikaite tufa columns in the Ikka Fjord in southwest Greenland represent a unique, permanently cold (<6°C), and alkaline (pH >10) environment (1) and have been shown to harbor a diverse bacterial community adapted to these conditions (2–4). A large number of bacteria from the ikaite columns have previously been isolated, and many of these produce extracellular enzymes active at high pH and low temperature (5, 6). One isolate producing high levels of extracellular proteolytic activity was characterized as *Arsukibacterium ikkense* GCM72^T^ (7). A close protease-producing relative (98.6% identity between 16S rRNA genes) was isolated from a series of alkaline ponds located in the McMurdo Dry Valley region in the Antarctica and named *Arsukibacterium* sp. MJ3. Both strains grow optimally at 15°C and pH 9 to 10 and produce extracellular proteolytic enzymes active at 5°C and pH 10. In this report, we describe the annotated draft genome sequences of these two strains of *Arsukibacterium*. The genome sequences are part of an ongoing research effort aimed at identifying new cold- and alkaline-active hydrolytic enzymes.

Genomic DNA was isolated from liquid cultures growing in standard R2 broth medium supplemented with 1% NaCl. Genome sequences were obtained by Illumina HiSeq paired-end sequencing of a short insert library. Assembly was performed using CLC Genomics Workbench (http://www.clcbio.com) for *A. ikkense*, resulting in a 4,127,744-bp genome assembly in 44 contigs (>400 bp) with an *N*_50_ contig length of 187,051 bp, and using IDBA (8) for *Arsukibacterium* sp. MJ3, resulting in a 3,746,433-bp assembly in 196 contigs (>400 bp) with an *N*_50_ contig length of 146,257 bp. The G+C content was 48.55%, and 46.02% for *A. ikkense* and *Arsukibacterium* sp. MJ3, respectively. Annotation of the genomes through the NCBI Prokaryotic Genome Annotation Pipeline resulted in 3,605 predicted coding sequences and 63 RNA sequences in the *A. ikkense* genome and 3,330 predicted coding sequences and 67 RNA sequences in the *Arsukibacterium* sp. MJ3 genome. The closest characterized relative of both strains is *Rheinheimera perlucida* type strain BA124 (DSM 15883), isolated from the Baltic Sea (9).

The genomes of both strains encode a number of putative extracellular proteases, including several serine proteases of the subtilisin type and numerous oligo-, di-, and exopeptidases, which may function in complete degradation of extracellular protein for nutritional purposes. Preliminary exoproteomic data on *A. ikkense* indicate that many of these proteases are secreted to the growth medium and may thus be responsible for the observed proteolytic activity at low temperature and high pH.

### Nucleotide sequence accession numbers. 

This whole-genome shotgun project has been deposited in DDBJ/ENA/GenBank under the accession numbers LAHO00000000 (*A. ikkense* GCM72) and LAHP00000000 (*Arsukibacterium* sp. MJ3). The versions described in this paper are the first versions, LAHO01000000 and LAHP01000000.
